# Altered stoichiometry and nuclear delocalization of NonO and PSF promote cellular senescence

**DOI:** 10.18632/aging.101125

**Published:** 2016-12-13

**Authors:** Ching-Jung Huang, Utsab Das, Weijun Xie, Miryam Ducasse, Haley O. Tucker

**Affiliations:** ^1^ University of Texas at Austin, Institute for Cellular and Molecular Biology, Department of Molecular Biosciences, Austin, TX 78712, USA

**Keywords:** cancer, cellular senescence, multifunctional proteins, stoichiometry

## Abstract

While cellular senescence is a critical mechanism to prevent malignant transformation of potentially mutated cells, persistence of senescent cells can also promote cancer and aging phenotypes. NonO/p54nrb and PSF are multifunctional hnRNPs typically found as a complex exclusively within the nuclei of all mammalian cells. We demonstrate here that either increase or reduction of expression of either factor results in cellular senescence. Coincident with this, we observe expulsion of NonO and PSF-containing nuclear paraspeckles and posttranslational modification at G2/M. That senescence is mediated most robustly by overexpression of a cytoplasmic C-truncated form of NonO further indicated that translocation of NonO and PSF from the nucleus is critical to senescence induction. Modulation of NonO and PSF expression just prior to or coincident with senescence induction disrupts the normally heterodimeric NonO-PSF nuclear complex resulting in a dramatic shift in stoichiometry to heterotetramers and monomer with highest accumulation within the cytoplasm. This is accompanied by prototypic cell cycle checkpoint activation and chromatin condensation. These observations identify yet another role for these multifunctional factors and provide a hitherto unprecedented mechanism for cellular senescence and nuclear-cytoplasmic trafficking.

## INTRODUCTION

Cellular senescence was originally defined as the point in which normal diploid cells cease to divide--generally after ∼50 cell divisions *in vitro* [1]. This phenomenon, also known as “replicative senescence”, can be provoked in response to DNA damage most prominent-ly by telomere shortening [reviewed in 2]. When a critical minimum length of telomeres is reached, their protective structure is disrupted [3]. An ensuing DNA damage response (DDR) is associated with the appearance of γ-H2AX and HP1γ positive foci and with DDR protein expression [4]. Communication between DDR-associated factors and cell cycle machinery amplifies the DDR signal into the senescence pathway [5]. In contrast, induction of “premature cellular senescence” occurs prior to the stage at which detectable telomere loss or dysfunction is observed [1,6].

Various conditions induce premature senescence in both cultured cells and *in vivo*. These include stress produced by inadequate culture conditions (eg, nonphysiologic oxygen; [7], oncogene-induced senescence [OIS; 8-10], and tumor suppressor loss-induced senescence [11,12]. A hallmark shared by cells undergoing replicative senescence and OIS is the critical involvement of the p53 and p16^INK4A^–RB pathways [13,14,15]. Increased expression and/or activity of p53 can be detected in senescence cells [2,16]. Retinoblastoma protein (pRb) is an active growth inhibitor in its hypophosphorylated form by binding to members of the E2F family of transcription factors [2,17]. In senescent cells, due to the high level expression of CDK inhibitors (eg, p16^INK4A^ and p21), Rb exists only in its active (hypo-phosphorylated) form (termed ppRb), thus preventing E2F-1 to activate genes required for re-entry into S phase [18].

In spite of the significant progress toward understanding the underlying mechanisms and complexities, there remains a need for better senescence biomarkers, as it is clear that no single one can reliably identify all senescent cells [1]. NonO/p54nrb [19,20] is a “multifunctional” protein for which numerous roles have been assigned [reviewed in 21,22]. These include transcriptional regulation through direct and indirect DNA binding and coordination of posttranscriptional elongation via interaction with the CTD of RNA polymerase II [23]. NonO has further been implicated in nuclear export [24] and in regulation of circadian rhythm [25-27]. NonO is typically found in hetero-dimeric complexes with Splicing Factor Proline/Glutamine Rich, PSF/SFPQ [28-30]. PSF is another multifunctional hnRNP that colocalizes with NonO within subnuclear domains termed paraspeckles [31,32]. Indeed, NonO, PSF and a third RNA recognition motif (RRM)-containing protein, PSPC1, are established “multifunctional” components of these subnuclear particles which collectively define the Drosophila behavior/human splicing (DBHS) family [21]. Paraspeckles accumulate as 10-30 particles/nucleus within all human transformed and primary cells [21,33].

Endogenous levels of tetrameric NonO:PSF complexes correlate with proliferation [30], whereas over-expression of PSF represses cell proliferation in cell culture and tumorigenesis in mice [34]. Notably, several reports implicate NonO and PSF in DDR [35-39]. For example, depletion of PSF induces chromosome breaks and fragmentation [35], whereas depletion of NonO or PSF delays DNA repair [35].

These findings led us to address whether NonO and/or NonO-PSF complexes may be linked to premature senescence. Here we report that both overexpression and reduction of NonO and PSF levels lead to senescence of transformed and primary human cells. Senescence induction correlates with paraspeckle-mediated, cell cycle-dependent relocalization from the nucleus to the cytoplasm, post-translational modifica-tion, hypophosphorylation of Rb, and imbalance of NonO:PSF heteromeric stoichiometry.

## RESULTS

### Overexpression of NonO reduces cell growth and senescence in transformed and primary cells

**Figure 1:**
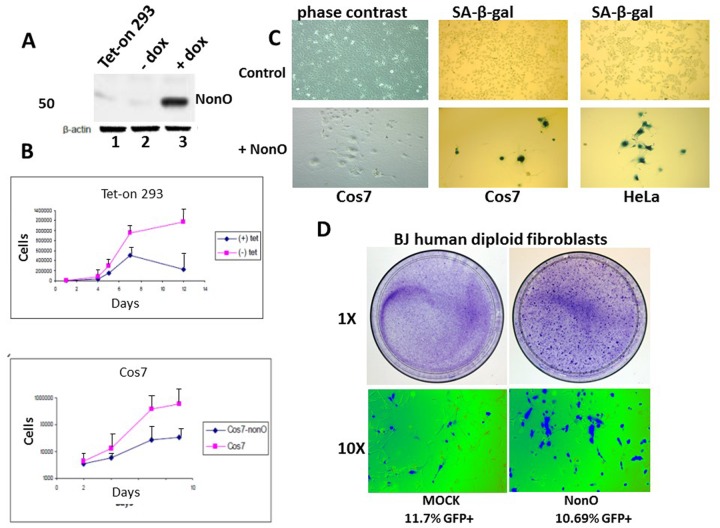
Overexpression of NonO promotes senescence of transformed and primary cells (**A**) Cell lysates from Tet-on 293 (lane 1), a clone stably transfected with NonO in the presence (lane 3) and absence (lane 2) of tetracycline, were confirmed for OE by SDS-PAGE/anti-NonO western blotting. Error bars represent average of 3 independent measurements. (**B**) Growth rate retardation following NonO overexpression. An inducible clone of Tet-on 293 (upper panel) or Cos7 (lower panel) was plated on day 0 at 10,000 cells/well in the presence (+) and absence (−) of tetracycline and harvested for cell counting as plotted as a function of time on a log scale. Error bars represent average of 3 independent measurements. (**C**) NonO overexpression promotes senescence of 293T and Cos7 cells, as measured by SA-β-gal staining. Empty vector (mock, upper panels); NonO overexpression (OE, bottom panels). Photographs are at the same magnification. (**D**) Overexpression of GFP-NonO promotes senescence of human diploid BJ fibroblasts. SA-β-gal staining indicated in blue of mock (left) and GFP-NonO (right) at day 12 following stable transfection/G418 selection. Magnification of 1 and 10X is indicated to the left of respective panels. Equivalent transfection efficiencies, indicated at bottom by %GFP+, were confirmed by flow cytometry analysis (not shown).

To test the effect of NonO overexpression on cell growth, we established lines that expressed tetracycline-inducible full length NonO (Fig. 1A). Following stable transfection of NonO into the 293 Tet-on cell line, clones were obtained but only rarely (eg, ∼1/35 hygromycin B resistant colonies). This suggested that leaky, uninduced NonO expression above the modest endogenous levels expressed in 293 cells may be toxic. Following Tet induction, clones exhibited elevated levels of NonO expression, reduced growth rates and ceased growth prior to reaching confluence (Fig. 1A, B). Similar if not greater growth reduction was also observed in Cos7 cells as well as several additional lines (Fig. 1B and Table 1).

**Table 1:** Senescence induction of immortal cell lines by NonO To assess the generality of NonO overexpression-induced senescence, population doublings (PDs) were determined for the indicated cell lines following stable transfection with PCR3.1-NonO; PCR3.1, mock, vector control.

Cell line	DNA transfected	No of clones lost proliferation/total number of clones	PD achieved prior to loss of proliferation
Cos7	pCR3.1-NonO	16/36	8-96
	pCR3.1	0/16	ND
CHO	pCR3.1-NonO	0/36	ND
	pCR3.1	0/16	ND
NIH3T3	pCR3.1-NonO	0/36	ND
	pCR3.1	0/16	ND
Cos7	pCR3.1-NonOAS	17/36	4-76
	pCR3.1-PSF	11/36	16-96
	pCR3.1-PSFAS	8/36	16-52

After serial passage, we observed unusual morphological changes in the recipient cell lines that overexpressed exogenous NonO. Cos7 and HeLa cells cultured at low density (Fig.1C, upper panels) adopted a flat enlarged morphology characteristic [1] of primary senescent cells and eventually ceased proliferation. While there was no observable apoptosis or necrosis-based death (Suppl. Fig. 1A), we observed strong senescence-associated -β-galactosidase (SA-β-gal) activity (Fig. 1C, lower panels). While SA-β-gal a generally accepted criteria for senescence, another reliable marker for true replicative senescence is the presence of senescence-associated heterochromatin foci (SAHF) [40]. As detected by anti-HP-1γ immuno-staining (Suppl. Fig. 1B), NonO over-expressing Cos7 cells revealed multiple SAHF foci, indicating that heterochromatinization was completed.

**Figure 2:**
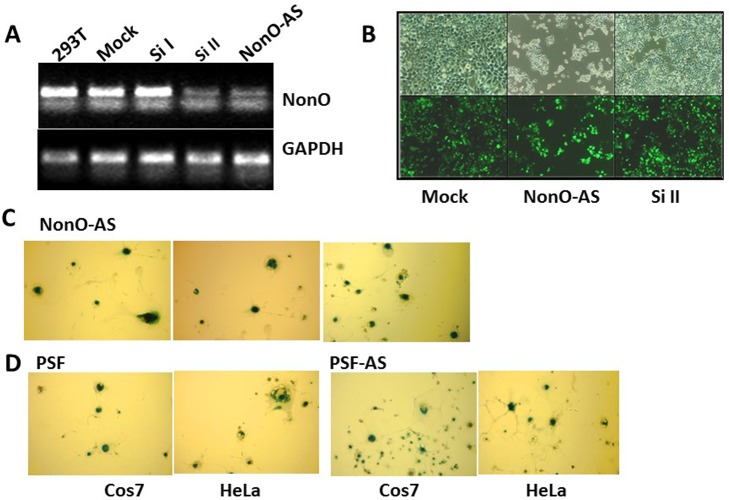
Knockdown of NonO or PSF induces senescence (**A**) Knockdown efficiencies for NonO siRNAs (si) and antisense (AS) cloned in pSuper-GFPNeo. Total RNA was isolated from 293T cells harvested 48 hr after calcium phosphate transfection with 10 μg either Si I or Si II silencing RNAs or with NonO AS DNA. Approximately 0.5 μg total RNA was reverse transcribed into cDNA which was analyzed by semi-quantitative PCR. (**B**) Growth reduction 72 hr post NonO knockdown as assessed by expression of pSuper-encoded GFP fluorescence microscopy at 1X and 10X magnification. (**C**) AS knockdown of NonO promotes senescence in Cos7 cells. G418-selected Neo^R^ clones of Cos7 or HeLa transfectants assayed at the indicated population doubling (PD) by SA-β-gal staining. (**D**) Overexpression (OE) or knockdown (AS) of PSF expression induces senescence in Cos7 or HeLa cells analyzed in bulk 96 hr following transfection.

Overexpression of NonO in low passage, human diploid BJ fibroblasts resulted in significant loss of growth and enhanced senescence within 12 days as assessed by either SA-β-gal (Fig. 1D) or by cell staining with 3-(4,5-dimethylthiazol-2-yl)-2,5-diphenyltetrazolium bromide) (MTT) (Suppl.Fig.2). Thus, both transformed and primary cells undergo rampant senescence when NonO levels are elevated.

### Down-modulation of NonO or PSF results in senescence

We employed both small interfering RNA (siRNA) and NonO antisense (AS)-containing vectors (described in Materials and Methods) to further analyze its requirement for senescence. 293T cells were harvested 48 h and 72 h after transfection with the silencing vectors (si1 and si2) and controls, and the knockdown efficiencies were determined to be sufficient (Fig. 2A). Unexpectedly, reduction of NonO expression resulted in the same phenotype as overexpression. As observed in Fig. 2B, cell growth was inhibited and strong SA-β-gal activity was detected (Fig. 2C).

NonO is typically found in a complex with PSF [28-30], and thus, we carried out similar modulation experiments for PSF. Western blotting confirmed that knockdown of PSF by siRNA or by antisense (AS)-mediated reduction had no effect on NonO levels (Suppl. Fig. 3). As observed for NonO, each of these constructs, regardless of whether they lowered or raised the endogenous levels of PSF, induced morphological changes in some of the G418-resistant clones (Table 1). These clones also were strongly positive for both SA-β-gal and SAHF, and they eventually ceased proliferation, whereas clones transfected with empty vector had normal growth rates and morphology (Fig. 2D, Suppl. Fig. 1B). As re-addressed below, these unanticipated results suggested that a stable NonO-PSF complex with a critical stoichiometry and concentration is important for growth of transformed cells.

### Cell cycle-dependent translocation of NonO to the cytoplasm within nuclear paraspeckles

**Figure 3:**
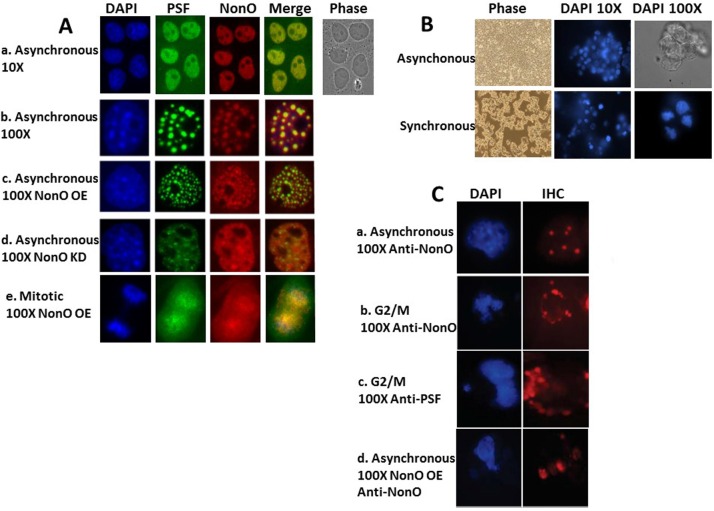
NonO-induced senescence is accompanied by cell cycle-regulated cytoplasmic localization within paraspeckles (**A**) Subcellular localization of NonO and PSF in Cos7 cells as determined by Immunostaining with anti-NonO or anti-PSF antibodies. (a,b) Endogenous NonO and PSF accumulation colocalize within the nucleus in asynchronous cultures; (c) OE of NonO in asynchronous cultures increases NonO:PSF paraspeckle nuclear localization; (d) NonO knockdown reduces NonO:PSF paraspeckle nuclear abundance; (e). NonO and PSF localize both in the nucleus and cytoplasm of mitotic cells within asynchronous cultures. Cell nuclei are visualized by DAPI staining. (**B**) Cell synchronization of ∼3 × 10^6^ 293T cells by sequential treatment with thymidine (16 h) and nocodazole (16 h) after an intermediate release step of 8 h as assessed by DAPI staining at 10X and 100X magnification. Untreated 293T cells (upper panels) grow asynchronously to confluency, whereas thymidine and nocodazole-treated cells (lower panels) loose adherence and arrest at G2/M with frequent condensed chromatin events. (C) Nuclear to cytoplasmic relocalization of NonO:PSF containing paraspeckles as identified by immunostaing with anti-NonO and anti-PSF antibodies. (a) Untreated asynchronous cultures; G2/M arrested cultures stained for NonO (b) and PSF (c); (d) Mitotic cells within asynchronous cultures follow overexpression (OE) of NonO.

Although alternatively spliced pre-mRNA isoforms of NonO and PSF have been observed [41], all forms characterized to date are exclusively nuclear [reviewed in 21] (Fig. 3Aa). A significant fraction of nuclear NonO and PSF have been shown to reside within paraspeckles [31-33]. After confirming these observations in asynchronous Cos7 cell cultures (Fig.3Aa,b), we observed that overexpression (OE) of NonO led to increased paraspeckle colocalization with PSF (Fig.3Ac), whereas knockdown (KD) of NonO resulted in loss of PSF signal (Fig. 3Ad). Thus, the majority of PSF nuclear accumulation/retention requires its association within NonO-containing paraspeckles. We further noted what appeared to be cytoplasmic relocalization of NonO and PSF as cells enter mitosis (Fig. 3Ae). To more formally test this hypothesis, we synchronized 293T cells at G2/M via nocodazole treatment (detailed in Methods and legend to Fig. 3B).

As expected, condensed chromatin events were frequent in the synchronized cells blocked in the G2/M phase and showed typical mitotic condensed chromatin within the nuclei (Fig. 3B). Following G2/M synchronization, NonO- and PSF-containing paraspeckles relocalized to the cytoplasm (Fig.3 Cb,c). Mitotic cells within asynchronous NonO overexpression (OE) cultures also contained delocalized NonO+ paraspeckles (Fig. 3Cd) These data suggest that senescence induction following up- or down-modulation of the PSF-NonO complex directly correlates with cytoplasmic relocalization of NonO and PSF. They suggest that this observed imbalance triggers not only relocalization of NonO and PSF to the cytoplasm but the mechanism that induces senescence (readdressed quantitatively with experiments to follow).

### A cytoplasmic mutant of NonO accelerates senescence

**Figure 4:**
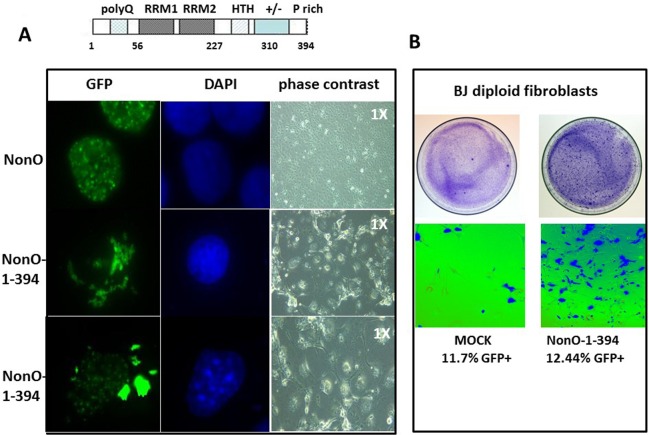
Truncation of the C-terminal 80 residues of NonO accelerates its cytoplasmic localization and senescence (**A**) Subcellular localization of full-length (GFP-NonO; upper panels) and C-terminal truncated NonO(1-394) clones (middle and lower panels) imaged 12 days following overexpression (OE) in Cos7 cells by fluorescence microscopy (left), DAPI staining (middle), and phase contrast (right). (**B**) Overexpression (OE) of GFP-NonO(1-394) accelerates senescence of BJ diploid fibroblasts. SA-β-gal staining of plates 12 days post transfection at 1X (upper panels) and 10X (lower panels) magnification. Equivalent transfection efficiencies indicated at bottom by %GFP+ were confirmed by flow cytometry analysis (not shown).

The above observations prompted the hypothesis that enforcing NonO into the cytoplasm of unsynchronized cells might accelerate senescence. Employing a series of NonO fragments fused to GFP, we determined that a construct containing residues 395-473 was sufficient for nuclear localization (Suppl. Fig. 4). Because residues 221-369 of NonO were previously shown [42] to be sufficient to retain PSF interaction, we chose a mutant which included this region along with its RRM domains but lacked the C-terminal 80 amino acids. As predicted, GFP-NonO (1-394) encoded a stable truncated protein that localized predominantly in the cytoplasm (Fig. 4A). Following transfection with GFP-NonO(1-394), most drug-resistant colonies underwent nuclear condensation and senescence at day 10-14, whereas most of the GFP-NonO wildtype transfected clones remained healthy (Fig. 4A). We also observed significant senescence induction in BJ diploid fibroblasts 7 days post-transfection (Fig. 4B). This is almost half the time required to observe senescence for full-length NonO under comparable levels (Fig. 1D, 4B).

### NonO-mediated senescence induction correlates with post-translational modification at G2/M

Previous studies revealed functional sites within NonO for both serine/threonine and tyrosine phosphorylation [reviewed in 21]. Notably phosphorylation of NonO within its coiled-coil C-terminal domain (T412, T430 and T453) and at Thr15 within its N-terminus by CDK1 both occur during mitosis with consequences on RNA binding occur during mitosis [43,44]. Nuclear envelope association of NonO also was observed in response to tyrosine phosphorylation [45].

**Figure 5:**
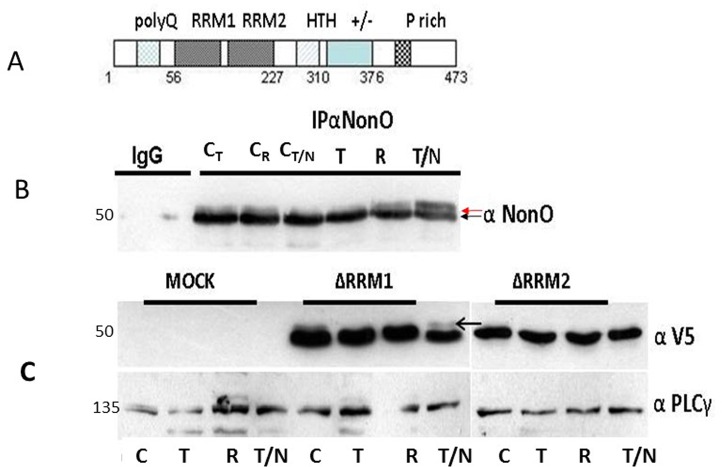
Posttranslational modification of NonO during mitosis (**A**) Schematic of NonO domains. polyQ, polyglutamine tract; RRM1 and 2, RNA Recognition Motifs; HTH, predicted helix-turn-helix domain; +/−, highly charged region; P, proline-rich. (**B**) The molecular weight of NonO (50 kD) is increased by post-translational modification at G2/M. Cells were lysed after 16 hr thymidine (T) pulse, then released for 8h (R) prior to incubation for 16h in nocodazole (N/T). NonO was immunoprecipitated with mouse anti (α)-NonO mAb and detected by Western blotting with rabbit polyclonal α-NonO; mouse IgG served as negative control. As controls, non-synchronized cells were lysed at the indicated time points in the same buffer (C_T_: 16 h thymidine; C_R_: 8h release; C_N/T_: 16 h nocodazole). A doublet is observed in control, but not in G2/M arrested cells. Modified NonO levels (upper arrow) increase between G1/S (thymidine-arrested) and G2/M (nocodazole-arrested) phases. (**C**) The NonO RRM2 domain is targeted by cell cycle-dependent modification. NonO deletion (Δ) mutants were analyzed under lysate and treatment conditions described above. The construction of NonO ΔRRM1 (lacking residues 87-160) and ΔRRM2 (lacking residues 160-227) is detailed in Materials and Methods. Protein loads in Western analyses were confirmed by anti-PLCγ (135 kD). NonO-deletion mutants were detected via their V5-N-terminal tags.

To determine whether NonO modification might correlate with senescence induction, we analyzed its mobility by SDS-PAGE in 293T cells synchronized at G1/S by thymidine block and at G2/M following nocodazole treatment. Fractions were collected at time points subsequent to release from both blocks and analyzed initially by western blotting. As shown in Fig. 5A, a doublet was observed in all control samples, whereas the upper, modified band (denoted by an arrow) was undetectable in extracts from thymidine-arrested cells. Thus, modified NonO levels (upper arrow) increase between G1/S (thymidine-arrested) and G2/M (nocodazole-arrested) phases (Fig. 5B). Deletion analysis (Fig. 5C) allowed us to map the modification to RRM2 (residues 160-227)—a region in which phosphorylation previously has not been detected.

### NonO-mediated senescence correlates with Rb hypo-phosphorylation and up-regulation of p53 and the DNA damage response

**Figure 6:**
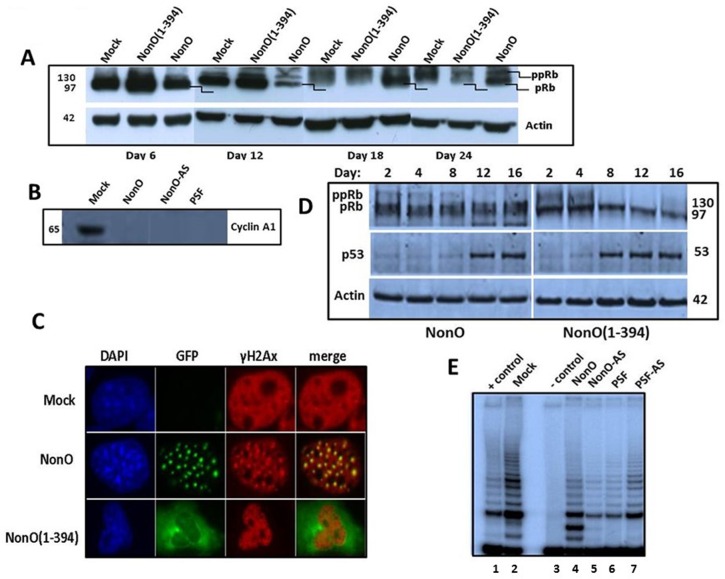
Overexpression of NonO activates G2/M arrest-associated signaling (**A**) Cos7 cells were stably transfected with either truncated GFP-NonO(1-394) or full length (FL) NonO, harvested at the indicated time points, and analyzed by SDS-PAGE/western blotting with anti-Rb mAb; anti-Actin mAb served as a loading control. (**B**) Expression of Cyclin A1 (lower panel) and p53 (upper panel) in GFP-nonO-transfected Cos7 cells. (**C**) Activation of DNA damage-associated punctate γH2AX formation 12 days following overexpression (OE) of FL or NonO(1-394). In pre-senescence nuclei, γH2AX clusters overlap with NonO-containing paraspeckles (middle panels), whereas in senescent nuclei, NonO(1-394) localizes to the cytoplasm while γH2AX foci are retained in condensed nuclei (lower panels). (**D**) Overexpression (OE) of NonO(1-394) (right panels) induces hypophosphorylation of ppRb to pRb and upregulation of p53 more rapidly than OE of FL NonO (left panels). Actin served as a loading control (bottom panel). (**E**) NonO or PSF overexpression (OE) (lanes 4 and 6) or antisense (AS) knockdown (lanes 5 and 7) do not activate telomerase activity in NIH 3T3 fibroblasts. TRAP assays as well as negative (−) and positive (+) cell extract controls (Lanes 1 and 3) are described in Materials and Methods.

Senescence is typically regulated through the control of several G1 checkpoint proteins, including p16, p21 and pRB [46]. To determine if NonO regulates this pathway, wildtype NonO and its cytoplasmic-specific truncation mutant NonO(1-394) were transfected into Cos7 cells maintained under G418 selection. Samples were tested at various time points for Rb phosphorylation status by western blotting. As shown in Fig. 6A, Rb phosphorylation remained unchanged at 6 and 12 days post-transfection. But on day 18, we observed a significant shift in the ratio from hyper-phosphorylation (ppRb) toward hypo-phosphorylation (pRb) in NonO transfected cells—a time coincident with senescence induction (Fig. 1B). The skewed ratio was maintained through 24 days of culture.

Overexpression of NonO(1-394) was accompanied by a higher Rb hypo-phosphorylation ratio at earlier time points as well as an increase in total Rb. By day 24, total Rb levels were significantly reduced. Perturbation of the Rb pathway was confirmed by loss of expression of the E2F1 target gene, Cyclin A1 following either up- or down-modulation of NonO and PSF (Fig. 6B).

In BJ fibroblasts, in which senescence induction is more facile than in transformed cells, we observed both rapid hypo-phosphorylation of Rb and induction of p53 (Fig. 6D). As predicted from the senescence induction kinetics, the NonO cytoplasmic mutant activated both G1/S and G2/M checkpoints more rapidly under similar levels of overexpression (OE) (Fig. 1D, 5B). Thus, both in transformed and nontransformed cells, cytoplasmic relocalization of NonO was accompanied by pRb to ppRb conversion and release of E2F1.

Histone H2AX becomes phosphorylated on serine 139 (thus termed, γH2AX) as a reaction on DNA double-strand breaks (DSB) [47]. Overexpression of NonO also resulted in a robust induction of punctate γH2AX formation—a quintessential indicator of DNA damage/G2/M arrest (Fig. 6C). Note that in pre-senescence nuclei, γH2AX clusters overlapped significantly with paraspeckle localization of NonO (middle panels). However, in senescent cells, in which NonO expression was primarily relocated to the cytoplasm, γH2AX foci remained in condensed nuclei (Fig. 6C, lower panels).

These data further suggest that perturbation of NonO or PSF levels activate G2/M arrest-associated DNA damage signaling.

### Telomere shortening does not contribute to premature senescence induction by NonO or PSF

We employed a TRAP assay [2] to investigate the telomere status during NonO and PSF-modulated senescence. As shown in Fig.6E, telomerase is still active in the senescent cells 20 days post-transfection. This result is consistent with our finding that a few clones rapidly reached senescence and ceased proliferation in just 4-8 population doublings (PD; the period of time required for a quantity to double in size or value; Table 1). The finding that senescence could be induced in such a short time period suggests that the telomeres within these senescent cells could not shorten to the threshold length, and thus could not contribute to senescence induction.

### The state of NonO heteromerization contributes to its export to the cytoplasm during senescence induction

It was unexpected that both over-expression and reduction of NonO or PSF resulted in induction of senescence. It was previously shown that NonO and PSF are capable of forming tetramers in malignant cells [30]. That result is consistent with subsequent studies by us and others demonstrating that NonO and PSF directly interact with very high affinity to each other and with themselves [28,30,48]. We suggested that NonO and PSF (∼55 and ∼100 kd, respectively) might form heterotetramers (2 × 50 + 2 × 100 ≅ 300kd) under the FPLC fractionation conditions we previously employed [28]. However, NonO:PSF complexes in the tetramer size range appeared to comprise only a minor fraction in HeLa cells, with the majority eluting as heterodimers [28].

Taken together, these results suggested to us that the stoichiometry of NonO:PSF complexes may allow NonO and PSF to fine tune their multiple functions. The data further predict that ectopic up or down-modulation of either member might alter this stoichiometry. Finally, we reasoned that such alteration in protein stoichiometry may be essential to the mechanism by which NonO, PSF, or NonO:PSF multimers are transferred to the cytoplasm during the initiation of senescence.

**Figure 7:**
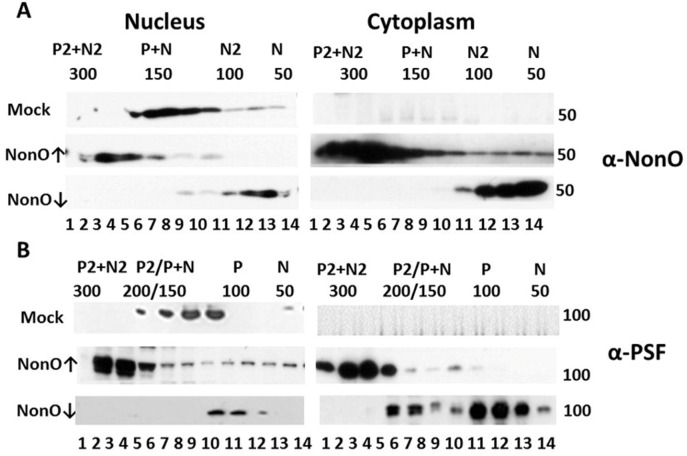
Perturbation of NonO-PSF stoichiometry induces nuclear to cytoplasmic relocalization during senescence induction Up- (indicated by ↑) or down (↓)-modulation of NonO levels leads to nuclear exit and altered stoichiometry of NonO (**A**) and PSF (**B**). Human BJ diploid fibroblasts were transfected with NonO (↑) or with NonO-AS (↓) and whole cell lysates were prepared just prior (day 8) to when senescence is observed (Fig. 1D). Nuclear and cytoplasmic extracts were prepared and then fractionated by Superose 6 FPLC under high salt conditions (400 mM KCl) to determine PSF (P) and NonO (N) elution profiles. Approximate masses in each fraction were assessed relative to the mobility of ferritin (440 kDa), catalase (232 kDa), lactate dehydrogenase (140 kDa), and albumin (66 kDa) run on parallel columns (Suppl. Fig. 6). Each fraction (indicated by numbers below the lanes) was concentrated and analyzed by Western blotting with anti-NonO (**A**) and PSF (**B**) antibodies. PSF (∼100 kDa) and NonO (∼50 kDa) elute either as monomers (P or N) or as part of a larger complex corresponding to homodimers (P2 or N2), heterodimers (P+N) and heterotetramers (P2+N2).

To test this hypothesis, human BJ diploid fibroblasts were stably transfected with either NonO or NonO-antisenese (NonO-AS) (detailed in the legend to Fig. 7 and in Materials and Methods) to achieve either Overexpression (OE; ↑) or Knockdown (KD; ↓), respectively. Just prior to senescence detection by SA-β-gal (day 8; Fig. 1D), cells were isolated, washed, and nuclear or cytoplasmic protein fractions were prepared. The resulting NonO:PSF complexes were subjected to FPLC equilibrated at relative high salt stringency (400mM KCl) to eliminate heterologous and/or nonspecific complexes. Molecular masses were estimated based on mobility of a mixture of protein markers (Suppl. Fig. 5).

As shown in the upper panels of Fig. 7, BJ fibroblasts express both NonO and PSF exclusively within the nucleus and fractionate at sizes corresponding to heterodimers (∼150 kD). Over-expression (↑) of NonO in BJ cells resulted in a skewing of nuclear abundance into a size range consistent with NonO:PSF heterotetramers (∼300 kd, Fig. 7A,B), along with emergence of complexes corresponding to homodimers (∼100 kD for NonO (Fig.7A) and ∼200 kD for PSF (Fig. 7B). Notably, a significant fraction of putatively heterotetrameric NonO:PSF was exported to the cytoplasm (↑ middle panels, Fig. 7A,B). In marked contrast, knockdown of NonO (↓ bottom panels) led to accumulation within both the nucleus and cytoplasm of monomeric NonO and PSF (∼50 and ∼100 kD, respectively; Fig. 7A,B).

While mass spectrometric analyses are required for formal proof, the data strongly suggest that NonO:PSF complexes other than heterodimeric are, at least in part, either retained in the cytoplasm or, more likely, exported into the cytoplasm where senescence induction is most strongly induced.

## DISCUSSION

Cellular senescence is a growth-arrest program that not only limits the normal lifespan of mammalian cells but prevents their unlimited cell proliferation in cancer. We found here that either overexpression or reduction of either of two interacting hnRNPs, NonO/p54nrb or PSF, promotes cellular senescence. Senescence was observed most rapidly and robustly following enforcement of normally exclusively nuclear NonO into the cytoplasm. Rationalization of these seemingly paradoxical data may be provided by stoichiometry. Following up or down modulation of NonO, the primarily heterodimeric NonO-PSF nuclear complex undergoes a dramatic shift in stoichiometry to heterotetramers or monomers, respectively. These complexes accumulate primarily within the cytoplasm coincident with DNA damage, cell cycle checkpoint activation and growth arrest.

NonO and PSF localization are dynamic; accumulation within nuclear paraspeckles being the primary site within non-stressed nuclei [33]. The localization and behavior of paraspeckles at different points within the cell cycle contrast with the behavior of many other nuclear domains. Unlike nucleoli and Cajal bodies which disassemble when cells enter mitosis [49-51], paraspeckles during daughter nuclei formation in telophase remain intact within the cytoplasm in perinuclear caps [33]. This, along with our observed G2/M checkpoint activation of p53 and γH2AX in diploid fibroblasts, further supports the notion that NonO and PSF cytoplasmic clusters derive from intact paraspeckles that accumulate during mitotic cell cycle arrest. Alternatively and potentially independent of a mitotic mechanism, a recently appreciated hallmark of senescent cells is a significant decrease in lamin B1 levels ([52,53]. Nuclear envelope integrity is breeched when accompanied by small chromatin herniation [54]. Either of the above mechanisms could account for the atopic cytoplasmic accumulation of NonO in the absence of nuclear export signals and provide a trigger for senescence. Consistent with this hypothesis, a truncated cytoplasmic form of NonO greatly accelerated senescence.

That PSF is also delocalized to the cytoplasm following modulation of it and/or NonO provides additional input into potential mechanism. A previous study [55] reported that a fraction of PSF is delocalized to the cytoplasm during mitosis following hyper-phosphorylation by Breast tumor Kinase (BRK). BRK is a non-receptor tyrosine kinase overexpressed in approximately two-thirds of breast carcinomas [56] as well as in some metastatic melanomas [57], renal carcinomas [58], and prostate cancers [57]—neoplasias in which either NonO, PSF or both have been implicated [21.^42^,^59^-^62^]. Phosphorylation of both NonO and PSF had been previously linked to cell cycle arrest [55,63]. We found that NonO undergoes post-translational modification--most probably phospho-rylation—at the G2/M phase of the cell cycle. We mapped this modification to RRM2, whose mutation was previously shown to result in severe localization and functional defects [32]. In addition to phospho-rylation, DBHS proteins can be modified by methylation, citrullination, SUMOylation and ADP-ribosylation [64-67]. However, as with phosphorylation, none of these modifications has been mapped to RRM2. Identifying the site and enzyme directed at RRM2 and its potential role in facilitating NonO-PSF mediated senescence remains a future goal of our studies.

NonO expression modulation also led to hypophoshorylation of Rb—another widely acknow-ledged senescence mechanism [reviewed in 13]. Rb hypophoshorylaton is typically associated with G1/S checkpoint activation by virtue of inactivation of the E2F family of transcription factors and subsequent recruitment of HDAC and other repressive factors that inhibit cell-cycle entry. By contrast, active E2F stimulates expression of multiple genes that play critical roles in the control of DNA replication and mitosis, including key drivers such as Cyclin A [68,69]. Thus, it is impossible to predict the explicit response to Rb inactivation and subsequent deregulation of E2F activity with reference to DNA replication and mitotic progression, because Rb-deficient cells can arrest in G2/M due to excessive genomic instability mediated via effects on chromosomal condensation [70,71]. Thus, our data cannot distinguish whether autonomous G1/S check point inhibition and failure to enter mitosis upstream of chromosomal condensation are coupled.

We demonstrated that loss of physiologic NonO:PSF heterodimer stoichiometry correlated directly with redistribution of both factors within the cytoplasm and with onset of cellular senescence. We appreciate that these results were obtained from in vitro manipulations of expression (ie, overexpression and knockdown). Mass spectrometric analyses are required for formal proof. However, that we detected these alterations prior to visual evidence of growth arrest strongly suggested more than a simple correlation with senescence induction. A precedent may be provided by the cyto-plasmic cycling of nuclear export adapters/receptors, which also appear to be regulated by their monomeric vs hetero-multimeric states [72]. Notably, Izumi *et al* (2014) identified NonO and PSF as U snRNA export stimulatory factors. However, NonO and PSF were shown to fall off the U snRNA export complex prior to its translocation through the nuclear pore [73]. Perhaps under those physiologic conditions, NonO:PSF hetero-dimers are retained within the nucleus, whereas the monomeric and tetrameric complexes are released. Another question posed by our data is how the different oligomeric states of nonO:PSF might provide functional properties distinct from their nuclear counterparts; for example, by providing exposed surfaces for interaction with cytosol-restricted effectors or posttranslational modifications. Identification of such interactions, which were purposely disrupted by the high stringency FPLC conditions required for assessing NonO:PSF stoichiometry, is an essential first step and is underway.

## MATERIALS AND METHODS

### Plasmids

pCR3.1-HA-NonO was constructed by excising full-length HA-NonO from pBS-HA-NonO1.4 vector with EcoRI/XhoI, and ligating it to EcoRI/XhoI digested pCR3.1 (Invitrogen). pBI-NonO2.4 was generated by excising full length NonO from p5.7NonO with XhoI and ligating it to SalI digested pBI (Clontech). GFP-NonO and deletion mutants were created by cloning a NonO cDNA into the pEGFP-C1 vector (Clontech). Two of the mutants, GFP-NonO 1-227 and GFP-NonO 228-473, were created by cutting NonO 1-227 and NonO 228-473 from pCR3.1-NonO 1-227 and pCR3.1-NonO 228-473, respectively, with XhoI/EcoRI and ligating them to XhoI/EcoRI digested pEGFP-C1. To construct GFP-NonO and all the other mutants, NonO fragments were generated by PCR using a 5′ primer containing a XhoI restriction site and a 3′ primer containing an EcoRI restriction site and template p5.7NonO. The fragments were cloned into pEGFP-C1 with XhoI/EcoRI sites. GST-NonO 1-227 and GST-NonO 228-473 were constructed by the exonulease recession method (Yang *et al*, 1993). To generate the NonO antisense construct – pCR3.1-NonOAS, full length NonO was excised from pSP72-NonO with XbaI/EcoRI and ligated to EcoRI/XbaI cut pCR3.1. PSF was cloned into pCR3.1. For the PSF antisense construct, pCR3.1-PSFAS, full length His-tagged-PSF was cloned into the Pmel site of pCR3.1 For NonO SiI and SiII antisense constructs, sequences were selected from genebank (sp|Q99K48|NONO_MOUSE) corres-ponding to nucleotides 487-508 (exon 5) and 1246-1266 (exon 9) nucleotides, respectively and cloned into pSuper.

### Antisense and RNAi for NonO and PSF

To make the NonO antisense construct (pCR3.1NonOAS), full length NonO was excised from pSP72-NonO with XbaI/EcoRI and ligated to EcoRI/XbaI cut pCR3.1. To generate the PSF antisense construct (pCR3.1-PSFAS), full length PSF was excised from pCR3.1-His-PSF with PmeI, then inserted back into PmeI digested pCR3.1. Constructs with antisense orientation were selected separately. For expressing short hairpin containing Si I and Si II of NonO, nucleotides 489- CGAACTGCTGGAAGAAGCC-507 (Si I) and 1248- AGGACCTGCCACTATGATG- 1266 (Si II) were separately cloned into pSuper.

### NonO RRM1 and RRM2 deletion mutants

The Gateway cloning system was employed for the cloning of NonO deletion mutants lacking RRM1 (deletion of RNA recognition motif 1) or lacking RRM2 (deletion of RNA recognition motif 2) in different expression vectors. Full-length coding sequence was amplified by PCR out of NIH 3T3 cDNA (mouse fibroblasts) and was inserted into the pENTR 2B entry vector between recombination boxes by conventional cloning. The RRM1 deletion mutant lacking amino acids 87-160 was obtained by double digestion with *Eco*RV (nucl:258) and *BSa*AI (nucl:480); the RRM2 deletion mutant lacking amino acids 160-227 was obtained by double digestion with *BSa*AI (nucl:480) and *Nru*I after inserting *Nru*I restriction site at the position 681 by site directed mutagenesis. The cloning in pENTR2B enabled transfer of the different constructs in pEF6-DEST51 and pDEST14 expression vectors through recombination.

### Cell culture and transfection

Transformed cells HeLa, Cos7, NIH3T3, HEK293 and CHO cells were grown in Dulbecco's modified Eagle's medium (DMEM) with 10% fetal bovine serum (FBS). Tet-On 293 cells were maintained in the same DMEM/FBS medium supplemented with 200μg/ml G418 (Gibco-BRL). For transient transfection, cells were grown on Lab-Tak chamber slides (Nunc) and were transfected with plasmid DNAs with Fugene 6 (Roche Molecular Biochemicals) according to manufacturer's instruction. Stable transfection was mediated by electroporation. Stable cell lines or clones were selected at ∼500μg/ml of G418 or 400μg/ml of hygromycin B (Calbiochem).

BJ human foreskin fibroblasts (Stemgent, Cambridge, USA) were cultivated in DMEM with high glucose containing 10% FBS, 2 mM L-glutamine, 1% penicillin/streptomycin, and 30 mM HEPES (Life Technologies, Darmstadt, Germany). Cells were kept at 37°C with 5% CO_2_ and media was changed every 3 days. Cells were passaged using trypsin/EDTA (0.04%/0.03%, PromoCell, Heidelberg, Germany). BJ fibroblasts at passage 7-9 were used for all experiments. For transfection, ∼1.5 × 10^5^ cells were plated per well of 12-well plate. Following overnight incubation at 37°C, transfections were performed with Lipofectamine 2000 and cell lines were maintained and selected by NeoR as described above.

### RNA Isolation and RT-PCR

For RNA purification, cells were first lysed and then homogenized through QIAshredder spin columns (Qiagen). Ethanol was added to the lysates which were then loaded onto RNeasy silica-gel membranes. Purified and concentrated RNA was eluted with water.

For RT-PCR, 25 μl reactions contained 1 μl cDNA diluted 1:40 and 0.1U polymerase). For each primer pair cycling and Tm were optimized (NonO: 35 x at 62°C / GAPDH: 34x at 55.8°C).

### Western blotting and immunohistochemistry

Cells were suspended in lysis buffer (50mM Tris-Cl [PH 8.0], 150 mM NaCl, 0.02% sodium azide, 1% NP-40, 100μg/ml PMSF, 1μg/ml aprotinin), and after 20 min on ice, lysates were cleared by centrifugation. Equal amount of lysates (determined by Bio-Rad Protein Assay, Bio-Rad) were run on 8-10% SDS-PAGE gels, and transferred to nitrocellulose membranes, and blots were developed according to standard procedures using ECL detection (Amersham). The following primary antibodies were used: monoclonal antibody NMT-1 for NonO (1:1000 dilution. Kindly provided by Dr. Moreland. Boston University School of Medicine), a mouse monoclonal antibody for Cyclin A (1:80 dilution. Oncogene), a mixture of monoclonal antibodies for Rb (1:50 dilution), and a monoclonal antibody for p53 (1:2000 dilution. PharMingen). Horseradish peroxidase-conjugated goat anti-mouse antibody (Amersham) was used as secondary antibody at 1:2500 dilution.

### Growth curves

Cells were plated in triplicate at ∼10^4^ cells/well on 12-well plates in DMEM/FBS. Cells were counted at the indicated times (Coulter counter). For the inducible cell lines, cells were grown in DMEM/FBS in either the presence or the absence of 1μg/ml of Doxycycline.

### Cell synchronization

Double thymidine-nocodazole block was used to arrest cells in mitosis. Cells were grown on 10 cm dishes one day before incubation. For initial G1/S block, medium containing thymidine was applied overnight (16 h) and removed on the next day. For G2/M block, cells were released for 8 h in normal medium and then incubated 16 hr with nocodazole. Mitotic cells rounded up and detached from the bottom of the dishes. Synchro-nization was monitored by microscopy and chromatin condensation was verified by DAPI staining. Imaging was performed with a Zeiss Axiovert 200M microscope and a Zeiss Axiocam camera. Filter setting was 01Ex 365/12 for Hoechst/DAPI staining and excitation was optimal at 365 ± 6 nm.

### Fluorescence microscopy and immunofluorescence

For immunofluorescence, cells were fixed with 4% paraformaldehyde for 30 min, permeabilized with 0.5% Triton X-100 for 15 min, incubated with blocking solution containing 3% bovine serum albumin and 0.2% gelatin in PBS for 15 min. Cells were then incubated with primary antibody diluted in blocking solution for 1 h at room temperature or 4°C overnight (1:500 dilution for NMT-1, 1:2000 dilution for mouse anti-SC35 [Sigma],1:1000 dilution of mouse monoclonal γH2AX (Ser-139), 1:1,000 (Upstate Biotechnology) followed by 1 h incubation with an anti-mouse second antibody conjugated with Rhodamine (Sigma). Cells were imaged on a Zxioplan fluorescence microscope (Zeiss) with a 40X plan NeoFluar objective. For direct fluorescence imaging, cells were fixed with 4% paraformaldehyde 48 h after transfection of GFP fusion constructs and stained with DAPI (50 ng/ml) for 5 min.

### Senescence-associated β-galactosidase (SA-β-gal) assays

Assays were performed as previously described [74]. Briefly, cells were washed once with PBS (PH 7.2), fixed with 1% glutaraldehyde in PBS (PH 7.2) for 30 min at room temperature, and washed once in PBS (PH 7.2) supplemented with 1mM MgCl_2_. Cells were then stained in X-gal solution (1mg/ml X-gal, 0.12 mM K_3_Fe[CN]_6_, 0.12 mM K_4_Fe[CN]_6_, 1 mM MgCl_2_ in PBS at PH 6.0) overnight at 37C (no CO_2_).

### MTT cell proliferation/viability assay

Suspension cells or trypsinized adherent cells were harvested by centrifugation at 500 x g for 5 min at 8°C. Following discharge of the supernatant, cells were resuspended at ∼5 × 10^6^/ml in 5 ml sterile cell culture medium, then serially diluted two-fold in triplicate prior to treatment with 10 ml MTT [3-(4,5-dimethylthiazol-2-yl)-2,5-diphenyltetrazolium bromide] (MTT) reagent; R&D Systems). Following incubation for 4 hours at 37°C, detergent-treated plates were incubated in the dark for 2 hr prior to absorbance reading at 570 nm.

### Telomerase assays

Telomerase activity was assayed essentially as described [75] using the TRAPeze telomerase detection kit (Intergen, NY). Cells were harvested by tryp-sinization, washed with PBS, and suspended in CHAPS lysis buffer followed by incubation on ice for 30 min. TRAP reactions contain 1X TRAP buffer, 50 mM dNTPs, γ-^32^P-ATP end-labled TS primer, TRAP primer mix, 2 u Taq polymerase, and 2 μl CHAPS cell extract in 50 μl. Each TRAP reaction mix was incubated at 30°C for 30 min to allow telomerase elongation of TS primer, followed by a 2-step PCR at 94°C/30 seconds, 59°C/30seconds for 27-30 cycles. PCR products were separated by electrophoresis on 12% non-denaturing polyacrylamide gels and visualized with a PhosphorImager.

### Fractionation of NonO-PSF nuclear and cytoplasmic complexes

Cells were harvested by trypsin-EDTA, collected by centrifugation, and washed 2X in ice-cold PBS. Cell pellets were resuspended in 5 packed cell volume of buffer F containing 20 mM Tris, pH 7.6, 50 mM 2-mercaptoethanol, 0.1 mM EDTA, 2 mM MgCl_2_, 1 mM PMSF supplemented with protease inhibitors (2 mg/ml aprotinin, 2 mg/ml leupeptin, 0,3 mg/ml benzamidin-chlorid, 10 mg/ml trypsin inhibitor) and incubated for 2 min at RT and for another 10 min on ice. NP-40 was added at a final concentration of 1% (v/v) and lysates were homogenized by passing 3X through a 20 G needle. Nuclei were pelleted by centrifugation at 600 g for 5 min at 4°C and supernatant containing cytoplasmic proteins was collected and stored at −80°C. Remaining nuclei were washed 3X in buffer F with 1% NP-40. Nuclei were stained with Trypan blue and micro-scopically examined for number, purity and integrity.

Extracts were loaded onto a Superose 6 (GE Healthcare) equilibrated in 400 mM KCl, 20 mM HEPES pH 7.9. 1.0 mM MgCl_2_ 0.5 mM EGTA and 10% glycerol. These high salt conditions were required to eliminate weakly interacting or spurious, heterologous proteins. Proteins were fractionated at a flow rate of 400 μl/min, and molecular masses were estimated based on mobility of a mix (Amersham Life Sciences) of ferritin (440 kDa), catalase (232 kDa), lactate dehydrogenase (140 kDa), and albumin (66 kDa) run on parallel columns. Collected fractions were precipitated with TCA, and the pellets were washed 2X with ice-cold acetone, dried and resuspended in 2X Laemmli buffer. Proteins were separated by 8% SDS-PAGE, transferred to nylon membranes and blotted with anti-NonO or anti-PSF under conditions described above.

## SUPPLEMENTARY MATERIAL AND fIGURE


