# Supplementary material for: Altered stoichiometry and nuclear delocalization of NonO and PSF promote cellular senescence
Source: Aging (Albany NY). 2016 Dec 13;8(12):3356–72. doi: 10.18632/aging.101125 (PMC5270673; doi:10.18632/aging.101125)
Supplement: Supplementary file 1 [file aging-08-3356-s001.pdf]

SUPPLEMENTARY MATERIAL

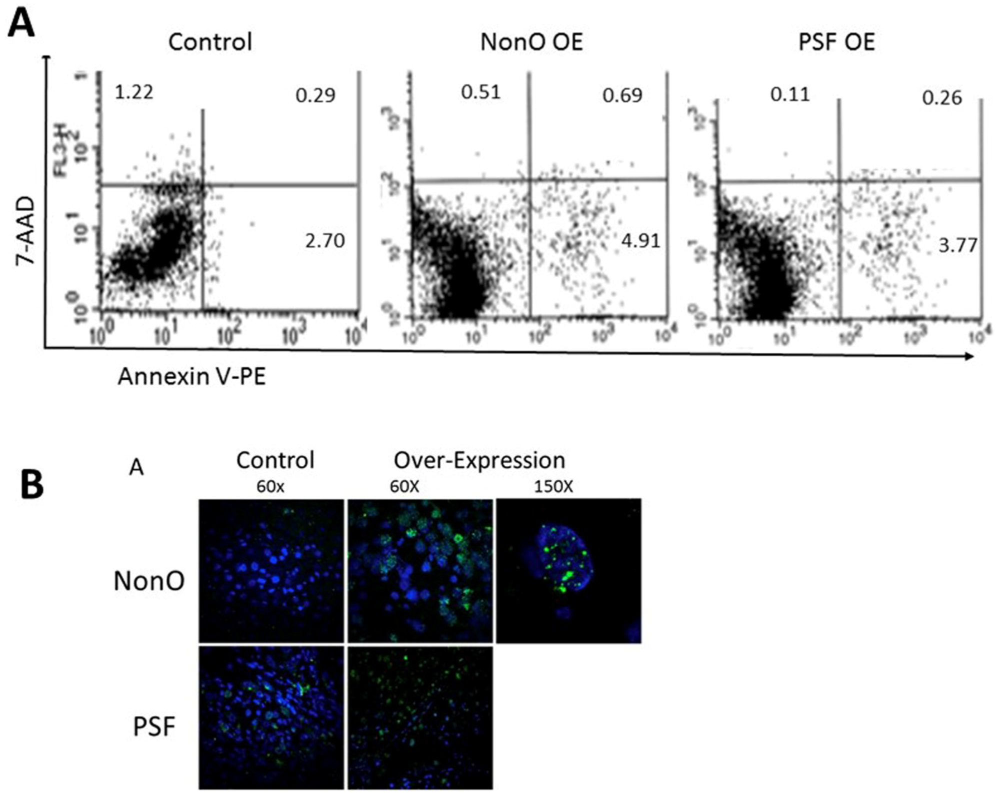

Supplementary Figure S1. NonO or PSF over-expression results in senescence-associated heterochromatin foci (SAHF) but not in apoptosis. (**A**) SAHF detection as detected by anti-HP-1γ immunofluorescence staining in Cos7 cells overexpressing NonO (NonO OE) or PSF (PSF OE). Foci aggregation and DNA damage was observed with FITC (green); nuclei were stained with DAPI (blue). Images were captured at 60× magnification with the exception of the right top panel which shows a magnified NonO overexpressing nucleus at 150X. (**B**) Neither NonO nor PSF overexpressing Cos7 cells die by apoptosis. Following stable transfection and growth (detailed in text and Materials and Methods), cells were assessed for apoptosis by dual-color FACS analysis. Annexin V stains apoptotic cells and the cell-impermeable fluorescent dye 7-AAD stains necrotic cells. Percentages of different populations are noted in the insets; 7-AAD+ PE-Annexin V+ apoptotic quadrant showed no difference between controls. The data shown are representative of 3 independent experiments.

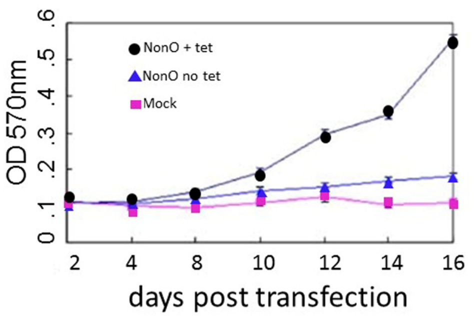

Supplementary Figure S2. Growth curve of senescence induction by NonO. Cos7 cells were left untransfected (MOCK ■) or were transfected with NonO in the presence (●) or absence (▲) of tetracycline as described in Materials and Methods. At day 2 post-transfection, cells were plated in 96-well plates (2000/per well) and stained at time points (days) following tet induction with MTT. Viable cell numbers were plotted as Absorbance at 570 nm. Plotted are the mean averages (**T**) of 3 independent experiments.

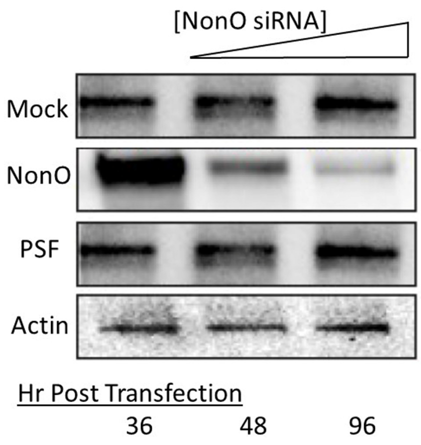

Supplementary Figure S3. NonO knockdown has not effect on PSF transcript accumulation. Total RNA was purified from nuclei of Cos7 cells transfected with vector or with NonO at varying time points following addition of tetracycline. The RNA was analyzed by semi-quantitative PCR from uninduced cells (MOCK) or from NonO transfectants for NonO and PSF levels. Actin served as a loading control. These experimental results are representative of three independent assays.

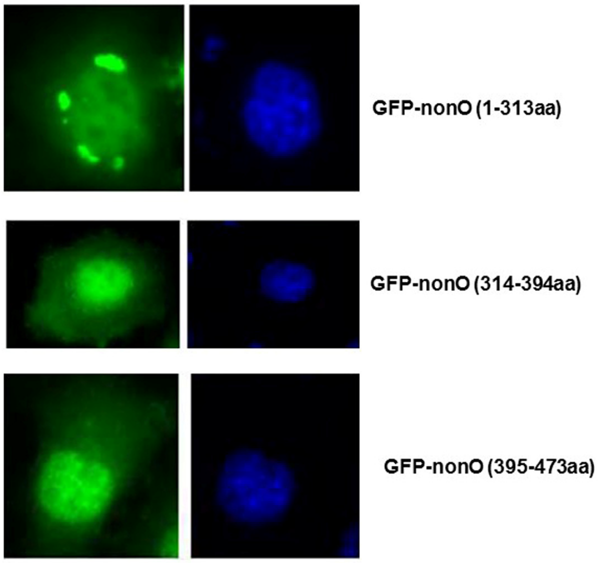

Supplementary Figure S4. Domains required for targeting NonO to the nucleus. Each of the indicated GFP-NonO constructs was transiently transfected into Cos7 cells. Forty eight hr post-transfection, cells were fixed and imaged for GFP (left panels) and DAPI (right panels). Error bars represent average of 3 independent measurements.

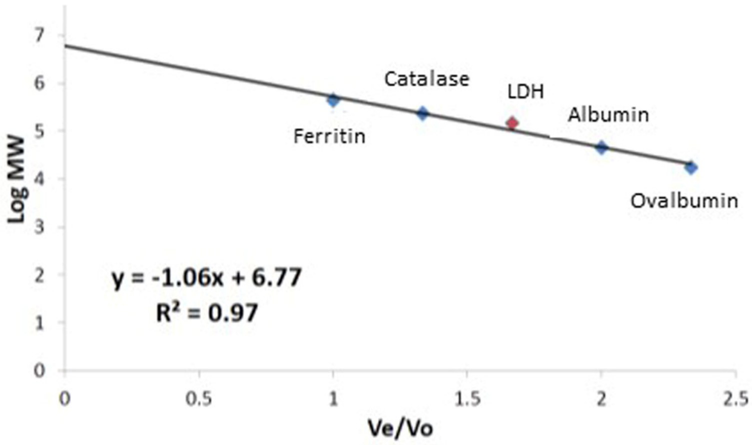

Supplementary Figure S5. Calculation of Homo- and Tetrameric NonO and PSF molecular masses. Molecular masses of NonO and PSF were estimated based on mobility during Superose 6 FLP chromatography of a mix (Amersham Life Sciences) of ferritin (440 kDa), catalase (232 kDa), lactate dehydrogenase (LDH, 140 kDa), and albumin (66 kDa) run on parallel columns. Linear relationship was observed between Log molecular weight and V_E/V_O, where V_E is the elution volume and V_O is the void volume.
